# Measurement Development for Japanese Clients’ Experiences during Adult Day Care Service Use (The J-AdaCa Tool)

**DOI:** 10.3390/healthcare8040363

**Published:** 2020-09-24

**Authors:** Takashi Naruse, Anthony G. Tuckett, Hiroshige Matsumoto, Noriko Yamamoto-Mitani

**Affiliations:** 1 Department of Community Health Nursing, Graduate School of Medicine, The University of Tokyo, Tokyo 113-0033, Japan; pb.hiroshige@gmail.com; 2 School of Nursing, Midwifery & Social Work, The University of Queensland, Brisbane, QLD 4072, Australia; a.tuckett@uq.edu.au; 3 Department of Gerontological home Care and Long-Term Care Nursing/Palliative Care Nursing, Graduate School of Medicine, The University of Tokyo, Tokyo 113-0033, Japan; noriko-tky@umin.ac.jp

**Keywords:** Adult day care, assessment, quality of care, aged care

## Abstract

Adult day care (ADC) is among the most common services in the Japanese long-term care context, but information on how such care is offered remains scarce. This study aimed to develop a measurement tool to assess the richness of clients’ experiences regarding their ADC service use. Through a collaboration with ADC administrators and staff, semi-structured interviews were conducted with three ADC clients (in one ADC agency), and a questionnaire survey (17 items about clients’ and their families’ experiences within ADC) was applied to 360 ADC clients (in 11 ADC agencies). Principle component analysis showed four factors regarding experience of ADC use: “Social participation”, “Hygiene and health”, “Exercise and eating habits”, and “Family support”. These positive experiences might be effectively provided if stakeholders refer to clients’ needs during ADC experiences, and their effective provision may relate to better care outcomes.

## 1. Introduction

With rapidly aging global populations, expenditure on long-term care (LTC) has increased; this share of the gross domestic product (GDP) has increased more rapidly than any other health care expenditure, and the annual growing rate during the 2005–2015 period was 4.6% across Organization for Economic Cooperation and Development (OECD) countries [1]. In Japan, the Ministry of Health, Labour and Welfare (MHLW) encourages the disabled older population to appraise and adopt relevant services depending on their needs [2]; it support such encouragement to help this populational group in reaching independence in the performance of their activities of daily living (ADL). Additionally, the MHLW has proposed to accumulate and analyze scientific data collected from LTC practices and their outcomes in Japan by scientifically valid indicators [3]. These indicators refer to the “whats” (i.e., the types of staff, locations where services are provided, supplies, etc.) and “hows” (i.e., care purpose, time length, methods, etc.) of adult day care (ADC) in Japan. Reportedly, OECD countries share preoccupations regarding the need for evidence-based support information in the LTC context, and the need for the proposals coming from such knowledge to be fulfilled [4]. Hence, the development of LTC measurement tools might contribute to the betterment of LTC systems in rapidly aging countries.

One of the major consequences of rapidly aging populations among OECD countries (Japan included) is the increased proportion of LTC recipients living at home [1]. Hence, in these countries, there may be a growing interest in ADC services/centers/agencies for the disabled older population. ADC is a generic term for aged care; it comprises building-based services that offer a wide variety of programs/amenities to the older people [5]. In Japan, ADC is a type of LTC service; in April 2018, the number of ADC clients was 1.13 million, and ADC was the most popular type of LTC service among the Japanese disabled population [6]. One international review demonstrated that providers had four general aims when providing ADC: (i) Providing social and preventive services, (ii) supporting clients’ continued independence, (iii) supporting clients’ health and daily living needs, and (iv) enabling family caregivers to take a break (from daily care) and/or continue with their employments [5]. ADC clients’ intervention-related benefits include improved physical, mental, and social function, exposure to comprehensive care, improved well-being, and the alleviation of family caregivers’ burdens [5,7,8].

A literature review on ADC effectiveness (2000–2011) revealed that ADC use relates to clients’ service-related experiences and to family caregivers’ respite from daily care [8]. Previous research emphasized these aspects; for example, results from a study describing ADC usage [9] were utilized to develop a scale to evaluate ADC services and processes underlying outcome performance [10]. Thus, it seems important to provide clients of ADC services with better experiences. However, owing to ADC clients’ and staffs’ heterogeneity, objectively demonstrating the benefits of ADC programs/interventions has been difficult [11].

In 2016, “The collaborative action research project for evaluation of ADC service quality” (CRAQ), a Japanese project from the Tokyo Council of Social Welfare and the University of Tokyo, was created. It was developed by researchers from the field of community health nursing and Japanese ADC clinicians, and it aimed to develop methodologies to measure/improve ADC service quality. To incite direct/immediate social changes in ADC community service agencies, the CRAQ adopted a collaborative project model, called community based participatory research [12]. It refers to a researcher–community partnership aimed at creating more equitable research processes between researchers and community members; this method increases the relevance of research questions, data, and programs devised/implemented for those directly affected by the studied problems/diseases [12].

**Figure A1:**
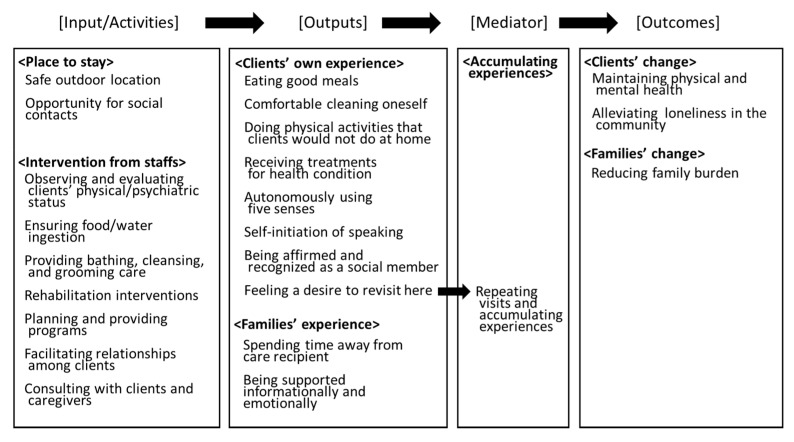
The logic model of Japanese clients’ adult day care service use [13].

At the beginning of the CRAQ, the logic model of ADC service use was described (Figure A1) [13]; it was a systematic chart with service input/activity elements and clients’ outcomes. The inputs had two aspects: place and intervention. This cited model showed that ADC services provided clients and their caregivers with various experiences, ranging from the fulfillment of fundamental care needs to psychiatric-/self-actualization, and that the continuous use of ADC services may result in the fulfillment of clients’ needs, subsequently allowing them to achieve their personal goals and improving the outcomes of ADC service use. This was the framework by which we outlined some of the potential constructs/items of the measure we aimed to develop in the study being reported here.

Based on the ADC logic model [13], the improvement of clients’/families’ experiences could improve service use outcomes. Correlatively, information on clients’/families’ richness of experiences during ADC service use can help identify areas in need of improvement (e.g., whether or not there should be efforts to increase opportunities for clients to exercise). Thus, to enable improvements in clients’/families’ experiences during ADC service use, having appropriate knowledge about how they use these services seems important. Based on this assumption, this study aimed to develop a measurement tool to assess clients’ richness of experiences regarding their ADC service use (Japanese clients’ experiences during adult day care service use: The J-AdaCa Tool).

## 2. Materials and Methods

This study was a part of the CRAQ activities. It comprised a qualitative (March to August 2019) and a quantitative phase (November 2019 to January 2020). We developed the measurement questionnaire methodology and items through semi-structured interviews and group discussions with ADC clients and staff, respectively. Then, a survey was conducted to collect data on clients’ ADC service use by using the aforementioned measurement tool. This study was approved by the concerned research ethics committee, Graduate School of Medicine and Faculty of Medicine, the University of Tokyo.

### 2.1. Collaboration Members in the CRAQ

Researchers collaborated with five staff (at one ADC agency) and eight administrators (at eight different ADC agencies). All collaborating agencies were located in Tokyo, and they were recruited through snowball sampling [14].

### 2.2. Development of Questionnaire: Methodology and Items

The ADC logic model [13] has 10 subcategories for clients’ experiences (8 separate ones, and 2 for clients and their family members) regarding ADC service use. Relying on the ADC logic model, the first author read the raw data of previous research in which the model was developed. Then, the development of the first questionnaire draft commenced. The first author extracted all words that described clients’ experiences from the raw data. Next, the first author proposed four scales to assess the extent to which older adults gained experiences from ADC service use, as follows: Amount of experience (five levels: high–low), intensity (five levels: strong–weak), frequency (five levels: frequent–infrequent) and satisfaction (five levels: satisfied–dissatisfied). At this point, we had yet to decide who would respond to these items (i.e., the patient, a family member, or an ADC staff).

To check the questionnaire validity, we conducted semi-structured interviews; three ADC clients (from one collaborating ADC agency) were included. The inclusion criteria were: (1) Had been using the current ADC agency for longer than three months and (2) being able to autonomously respond to the researcher’s questions. The agency administrator introduced the first author to potential participants, and the researcher asked for their participation. After individual written consents were obtained, individual interviews were conducted.

The first author asked questions about their experiences while they were at the ADC agency to check the coverage (i.e., the extent to which their experiences during ADC service use were included in the item contents or not) and wording of the first questionnaire draft. Interviews lasted for about 60 min, and all interview transcripts were typed out verbatim. Then, the first author carefully read through the transcripts and modified the questionnaire based on participants’ feedback on the coverage and wording of the first questionnaire draft. This interview only aimed to check the coverage and wording of the questionnaire draft, and the transcript data were referred to in raw data.

After the first questionnaire draft was modified, the researcher discussed its contents with all collaborators to develop the final questionnaire draft. We acknowledged that more than a third of the ADC clients did not have family caregivers, and more than half of them were expected to have some degree of dementia. Therefore, it seemed inappropriate to evaluate clients’/family members’ experiences based solely on clients’ responses to the questionnaire. Based on this, we designed the final items (Table 1) taking into account that ADC staff would be the respondents, ensuring that their information would be useful and sufficient for the identification of clients’ experiences.

### 2.3. Quantitative Survey Phase

Chart surveys using ADC staff administering questionnaires were conducted in 11 ADC agencies in Tokyo. The first researcher provided a face-to-face explanation of the survey for 38 participants, who were staffs from ADC agencies, participating in a routine skill-up seminar. The seminar was organized by the Tokyo Council of Social Welfare (i.e., a social welfare organization) in 2019. The ADC skill-up seminar is a self-study seminar for ADC (belonging to the Tokyo Council of Social Welfare) service staff aimed at improving care quality; it takes place about once every three months. Seminar participation is voluntary, and the usual numbers of participants shift around 30–60 staff in 20–40 ADC agencies. These seminars are usually announced to staff through the ADC by the organizer (e.g., pamphlet distribution or email is sent to ADC administrators, who then inform ADC staff about the event). Moreover, participation is free—the corporation running the event covers event costs. In total, 15 agencies (including the six already collaborating agencies) within the seminar showed intention to participate.

The survey among clients was conducted between September 2019 and March 2020, in Tokyo, Japan. Study instructions, consent forms and questionnaires were mailed to administrators of the 15 aforementioned agencies. If administrators answered the consent form, it was implied that the ADC agency agreed to collaborate. Regarding questionnaire application, administrators chose one usual typical service day (chosen based on their own administrative settings) in which they would conduct the survey in their respective ADC agencies. Administrators explained the purpose and methodology of this study to their respective ADC clients. The inclusion criterion was clients who had already been using the ADC for three months or longer. To ensure participants complied with this criterion, one ADC staff (i.e., either the chief staff in the respective center or a staff who had thorough knowledge of clients’ current status) gathered data about clients who visited their agency on the day of survey application; this data was gathered from chart information and administrators’ subjective assessments. Complete consent forms and questionnaires were mailed back to the researcher.

Since principal demographic data (except for names) were collected from clients’ charts by the ADC staff, these clients were not required to provide a written consent form.

#### 2.3.1. Measures

The examined variables included clients’ demographic factors, ADL and dementia severity. Demographic factors included age, gender, living condition, and frequency and history of ADC service use. All items included in the final questionnaire were checked for face validity and ease of answering by all collaborating members.

Regarding the ADC clients’ ADL, we created original items by referring to the Katz index [15,16]. This index is used to assess people’s functional status by analyzing their ability to independently perform their ADL; it comprises six types of ADL independence (performance adequacy in activities regarding bathing, dressing, toileting, transferring, continence and feeding). In our study, we measured clients’ difficulty in performing three ADLs: toileting, standing up, and feeding. Each item was created specifically for each activity, but all asked about the extent to which one required assistance to carry out the corresponding activity. Participants answered these items based on a 2-point scale: “need assistance” or “independent”. We included only three ADLs in an effort to make the task less onerous for the respective ADC staff.

Dementia severity was measured by two items of the Inter Resident Assessment Instrument(Inter-RAI)(home version) from a Japanese guidebook (the original guidebook has 7 items) [17]. The Inter RAI is used to assess people’s functioning in home care settings and the quality of their care, and the guidebook is used to assess people’s problematic behaviors related to dementia. From the Japanese guidebook, we included items on socially inappropriate behavior and refusing care; these two were chosen by research collaborators based on their feedback that these were common problem behaviors among ADC clients (while the others were not recurrent), and we chose only two questions in an effort to make the task less onerous for the respective ADC staff.

In the Inter RAI, the care provider responds to items (regarding subjects’ problematic behaviors frequency) based on a 4-point scale: (The behavior occurred) “In all three days”; “One or two days within the last three days”, “Sometimes, but not within the last three days”, and “Not at all”. Given our aims (examine problematic behaviors frequency in ADC settings), the 4-point scale was modified to: “Every time this client came to the ADC agency”, “Once every two times this client came to the ADC agency”, “Sometimes, but less than once every two times this client came to the ADC agency”, and “Not at all when this client came to the ADC agency”.

Clients’/families’ experience frequency during ADC service use was measured via the original 17 items developed in the qualitative phase and enhanced by the pre-tests (described below). The ADC staff responded to the items (based on their self-assessments) on a 4-point scale: 1 = Not at all, 2 = Rarely, 3 = Once every two visits, 4 = Every visit.

Prior to data collection, the final questionnaire draft was pre-tested by a small group of CRAQ collaboration members. Between May and June 2019, two members performed a pre-test with a sample of 20 ADC clients in one ADC (staff A and B in ADC-X in the Tokyo area). Afterwards, the first author, staff A and B, and one administrator discussed the final questionnaire design. Before finalizing the questionnaire, another pre-test was performed; in July 2019, three staff (staff B, C, and D in ADC-X) independently administered the questionnaire to 20 clients for a total of 60 clients. The collected data were evaluated by one administrator (administrator X, who usually played a supervisor role for staff in ADC-X) and one member of staff (staff A, the most experienced in ADC-X). After they confirmed the quality of the answers, the questionnaire was modified based on their assessments. Similar to previous procedures, the finalized questionnaire was checked by all collaborators prior to the final survey application. For each client, the staff average questionnaire completion time was around 7 min.

#### 2.3.2. Data Analysis

Statistical analyses were conducted by SPSS version 24 (IBM Japan, Ltd., Tokyo, Japan). Frequency and mean were calculated on all clients’ demographic variables and each experience items. Then, principal component analysis with varimax rotation was used to summarize the empirical dimensions of the items. Based on the scree plot, a four-factor solution was used, and the four factors accounted for 65.8% of the variance. A two-factor solution could be adopted with 41.3% variance, but the fourth factor was considered as showing “caregiver’s experiences”, and it was determined to be an important factor in the measurement tool for improving ADC experience when the first author and CRAQ had a discussion about data analysis. Then, a four-factor solution was adopted. Based on a previous study [10], items with factor loading >0.45 were included (Table 2). To create a factor composite score, load weighted responses (factor loading scores × item response) were calculated for each individual/item. The reliability of the measurement was determined using Cronbach’s alpha for each four factors with load weighted responses.

## 3. Results

Among the 15 ADC agencies that were interested in participating in this research, we collected data from 11 participating ADC agencies, and 360 clients met the inclusion criteria. The other four agencies were interested in participating in this research in March 2020; nonetheless, owing to the spread of the Coronavirus disease–2019 in Japan [18], the researchers chose to cancel their participation. The cancellation was communicated in February 2020. To avoid confusion among ADC staff and clients, all surveys of these agencies were cancelled.

### 3.1. Characteristincs of Participants

Clients had a mean age of 85.4 (standard deviation (SD) = 6.8; range: 55–102) years old. In total, 263 clients (73.1%) were women, and 105 (29.2%) lived alone. About 80% showed no problem with dementia in either of the two analyzed behaviors; totals of 80.6% in “socially inappropriate behavior”, and 81.4% in “refusing care”. Regarding ADL, 31.7% of the clients needed assistance with toileting, 29.4% when standing up (29.4%), and 13.9% when feeding. Averagely, clients visited the ADC 2.6 (SD = 2.0: 1–6) times a week and had used it for 4.1 (SD = 3.8: 1–20) years.

### 3.2. The Frequency of Experiences of ADC Service and Principal Component Analysis

Table 2 and Table 3 include the frequency of experiences of ADC service use and the results of the principal component analyses. The 4-point Likert-type scale used in this instrument allowed for scores ranging 1 to 4 (“Not at all” to “Every visit”). Mean scores ranged 2.08 to 3.58, indicating that each experience occurred on a rare to frequent basis.

More than 70% of the clients reported that they experienced the following items every visit (4): 71.17% for “Eating more than usual (Ex1)”, 71.9% for “Exercising more than enough (Ex5)”, 72.8% for “Feeling like revisiting here (Ex14)”, and 72.5% for “Recognizing one’s own place in society (Ex15)”. Less than 10% reported “Not at all” for these previously cited items.

The clients reported “Not at all” at higher ratios in the following items: 44.4% for “Family members being supported (Ex17)”, 40.6% for “Being advised in the amount of ingested food (Ex3)”, 39.7% for “Receiving treatment (Ex8)”, 39.4% for “Receiving explanations about one’s health conditions (Ex9)”, “Cleaning oneself (Ex4)(30.3%)”, and 28.1% for “Family member being away from the care recipient (Ex16)”.

The sum of the responses rated as 1 and 4 accounted for more than 50% of the total responses for all items, and more than 75% of the clients reported either “Every visit” or “Not at all” for the following items: 78.4% for “Being advised in the amount of ingested food (Ex3)”, and 83.7% for “Family member being away from the care recipient (Ex16)”.

The factors from the principal component analysis were named through discussion among the collaborating members of the CRAQ. The first factor was named “Social participation” (eight items), comprising items on behaviors within group settings and clients’ reactions to others (e.g., other clients). The sum of its loading squares was 25.4%, which exceeded those of the other three factors by 10%.

The second factor was named “Hygiene and health” (three items), comprising items on medical/nursing interventions, hygiene and nutrition interventions; it explained 15.9% of the variance.

The third factor was named “Exercise and eating habits” (three items), comprising items mainly on exercise, but it also had some on eating habits; it explained 14.1% of the variance.

The fourth factor was named “Family support” (two items), comprising items on family members’ experiences; it explained 10.4% of the variance. Only one item, “Eating more than usual”, loaded onto two factors—the first and third factors.

The reliability of the measurement was determined using Cronbach’s alpha, and the four factors showed α scores of 0.890, 0.765, 0.744 and 0.757, respectively. The Kaiser–Meyer–Olkin measure of sampling adequacy was 0.832, showing strong sample validity. The distribution of all item responses was shown to collaboration members in the CRAQ, and it met their clinical expectation. The members agreed that the items will be able to show the distribution of clients’ experiences in Japanese ADCs.

## 4. Discussion

This study aimed to develop a measurement tool for clients’ experiences regarding ADC service use to support quality improvements during ADC. Our study resulted in a 17-item questionnaire that can be used by ADC staff without complication and in a short period of time (i.e., 7 min on average).

### 4.1. Response Distribution

Two types of experiences can be found based on item responses: those common to ADC clients and those specific to clients’ needs. Specifically, four items were experienced every visit by more than 70% of the clients; thus, they may refer to common situations and activities during ADC. Moreover, six items were never (“Not at all” in the measure) experienced by more than 25% of the clients; still, 20% of the clients responded that they experienced these same six items every visit. This suggests that ADC clients are exposed to different experiences, and the overall effectiveness could differ between each client. When evaluating ADC outcomes among LTC practice [3], clients’ objectives and daily experiences should be included.

One item, which related to cleaning one’s self, had a missing answer ratio of 7.5%, which was higher than that for all other items. Upon reflection, we noticed that “cleaning” in the item referred to brushing teeth and face-washing, so we think that the answer may have been troublesome for ADC staff. Generally, ADC services in Japan provide cleaning services that relate to bathing, teeth-cleaning and hair styling [19]. Thus, this item may be improved by including a wider range of cleaning services in the definition of “cleaning” in the question’s wording.

### 4.2. Component Factors

#### 4.2.1. Factor 1: Social Participation

Social participation can be defined as a person’s involvement in activities that provide interaction with others in society or the community [20]. Hence, we chose this name for the first component because its items described situations in which clients spent a few hours surrounded by other clients and staff—namely, social contact inherent to the ADC. In this factor, the average raw scores for each item were higher than 2.9; namely, ADC clients experienced all social situations contained in these items once every two visits or every visit. Hence, social participation seems to be the most common experience for most ADC clients.

The aforementioned higher scores in this factor mean that the ADC clients in our sample engaged in social interactions with some relevant frequency; hence, we can infer that higher scores in this factor relate to beneficial outcomes with regards to people’s social participation. Indeed, previous literature acknowledges that social participation improves clients’ physical and mental health and promotes positive social relationships [21]. Further corroborating this, the maintenance/development of social relationships and access to the world through ADC activities was determined to improve clients’ psychosocial well-being [22]. Additionally, social participation makes old people more stimulated, confident and content [23]. In corroboration of this, this increased social participation among ADC clients appeared to encourage increased activity with newly-met ADC friends [24], and/or with ADC clients’ pre-existing social networks outside the ADC setting [23]. Furthermore, the Japanese Council of Social Welfare showed, through an interview survey among ADC users, that their social interactions with other ADC clients was expected to have an empowering effect (e.g., it stimulates the clients and restores their confidence [25]).

#### 4.2.2. Factor 2: Hygiene and Health

The Hygiene and health factor related to clients’ experiences with professional care or to supportive environments for the performance of their ADL. Particularly, this factor included items related to clients’ experiences with observations/interventions from nursing staff (e.g., nursing treatment assessments, medical status explanations, and nutritional advice). “Receiving assessment” was common, but the other items were less common, namely, they related to clients’ specific needs. ADC nurses might first provide general nursing assessments common to clients, and then provide specific interventions to clients who need them. The greater quantity of experiences in this factor should be expected to improve outcomes in clients with medical needs specifically, and the higher score might implicate the better ADC service among them. However, we cannot confirm whether less experience indicates “not enough experience among people with needs” or “less experience because the person does not need it”. The score might not reflect the service quality among clients without medical needs because they do not need much experience with direct hygiene intervention. In Japan, a nationwide survey found that 16.8% of the users needed medical intervention while staying at an ADC [19]; the survey showed that the most common needs were, in order: medication management, blood glucose monitoring and/or insulin injection, and oxygen therapy.

#### 4.2.3. Factor 3: Exercise and Eating Habits

The exercise and eating habits factor included three kinds of experiences with exercise and diet programs aimed at preventing disability development. Behaviors related to this factor are expected to prevent diseases/injuries before they even occur, or at least slow their progress. On this topic, a study reported that ADC attendance prevented daily problems that resulted from declined physical or emotional functioning [26].

Specifically, the item “Eating more than enough” was involved in both the Social participation and Exercise and eating habits factors. Previous studies show that this association makes sense: social isolation and malnutrition are issues that co-occur with a certain frequency among the older persons [27]. The two are closely linked, since eating with others can improve energy intake compared to eating alone [28]. In the ADC, social and nutritional services are, usually, simultaneously delivered via shared mealtimes [29]. Therefore, mealtimes may be a point of intervention for delivering both social engagement and nutrition to ADC clients.

#### 4.2.4. Factor 4: Family Support

The Family support factor included two items related to the experiences of clients’ family members during ADC, and it had the smallest factor loading (10.4%). Nonetheless, a specific situation could have contributed to such low loadings: one of the items was about family members’ spatial separation from home, which allowed for family caregivers to spend time away from the care recipient. Given that about 29% of the participants lived alone, this type of experience was not applicable to our whole sample. Previous studies show that caregiver burden can be alleviated through the provision of respite from their daily caregiving activities and of individual support [30]; hence, ADC service use is expected to reduce a caregivers’ burden and improve their health. Gaugler developed a self-reported measurement tool of ADC service processes and records the family caregivers’ responses [9,10]. Currently, we believe this may be the best method to measure caregivers’ experiences regarding ADC service use, as it comes from their perspectives.

Moreover, our results indicated that clients’ and family caregivers’ experiences can be independently assessed, so we believe that future research could make use of Gaugler’s (2014) measurement tool to directly assess caregivers’ experiences during an ADC placement [9].

### 4.3. Clinical and Research Implication

The biggest challenge of this study was trying to describe clients’ experiences in the ADC setting as measurable items. Nonetheless, we were able to explain the meaning of each type of experience through four factors we developed. These aforementioned factors corroborated themes in previous literature reviews about ADC service processes [7,8], and corroborated the four general aims of ADC services proposed by Orellana [5].

Specifically, in Japan, explaining the “whats” and the “hows” of ADC services (occurring in every ADC use) from the clients’ perspective is perceived to be a difficult task [25]. Such difficulties may hinder the understanding of disabled older persons who have never used an ADC service, especially regarding the benefits of ADC service use. Thus, our CRAQ team, which included ADC professionals, tried to make the item descriptions and the factor names as simple and easy to understand as possible, so that most ADC professionals and clients can respond to the instrument effortlessly. Thus, we believe that the developed items could be helpful for ADC staff when they try to explain the meaning of ADC clients’ experiences to disabled older persons and their families. Nonetheless, we highlight the need for further examinations about the usability, reliability and validity of the measure.

Additionally, some health care quality assessments use patients’ subjective experiences as a quality indicator, and our questionnaire can eventually provide this type of information as well. Our study showed that 17 kinds of ADC experiences were integrated into four factors, and higher ratings in all factors may prove beneficial to clients’ and their families’ future physical/psychiatric/social health. Additionally, based on the results of the factor loadings, we believe it may be possible to create integrated composite scores for each client; in turn, these can show clients’ degree of exposure to these four kinds of ADC services. Thus, future studies are warranted to examine the predictive associations between these factor composite scores and future health statuses among ADC clients. With such information, people’s scores in the factors of our questionnaire may be used to measure the quality of ADC services. Finally, such knowledge may contribute to the betterment of ADC services.

### 4.4. Limitations

Notwithstanding, this study has three clear limitations. First, the possibility of social desirability bias among ADC staff; namely, they could have over-estimated clients’ experience frequencies during ADC service use. Second, the possibility of sampling bias; the number of collaborating agencies was small, and, through the study procedures, ADC administrators might have included their self-interests regarding clients’ experiences/quality evaluation. This is unconnected to the spread of Coronavirus. That is, clients’ experience frequencies in our results may be higher than those of general ADC clients. We should improve the items so as to prevent social desirability bias, and increase the number and diversity of agencies so as to represent general ADC agencies.

Therefore, further research should develop questionnaire items through collaboration with family caregivers, examine the component structures with data from broader and more representative samples, and item test–retest/inter–rater reliability tests should be conducted to provide information on questionnaire validity and reliability.

## 5. Conclusions

Our principle component analysis showed four component factors regarding ADC clients’ experiences during ADC service use. ADC clients’ experiences were explained by three factors: “Social participation”, “Hygiene and health” and “Exercise and eating habits”. The separate fourth factor was “Family support”. Our discussions showed that the provision of positive experiences regarding these three factors may be ensured if the relevant stakeholders refer to clients’ needs regarding ADC service use when conducting this type of care. Moreover, ensuring the provision of positive experiences can relate to better care outcomes. Finally, future research should modify the items based on family caregivers’ experiences and examine their reliability/validity coefficients.

## 6. Patents

This section is not mandatory, but may be added if there are patents resulting from the work reported in this manuscript.

## Figures and Tables

**Table 1:** Questionnaire items about clients’/family members’ experience regarding adult day care service use.

Item	Item Name	Definition
Ex1	Eating more than usual	Whether or not a client eats more in the ADC agency than he/she usually does in his/her home setting.
Ex2	Comfortable mealtime	Whether or not a client enjoys others’ presence or is comfortable around others during his/her mealtime.
Ex3	Being advised in the amount of ingested food	Whether or not a client receives advice and adjustments regarding his/her nutritional input to enhance it.
Ex4	Cleaning oneself	Whether a client is able to autonomously, safely, and comfortably take a bath or whether he/she receives care to keep him/herself clean.
Ex5	Exercising more than enough	Whether or not a client exercises appropriately and more than enough because the ADC staffs, programs, or equipment support it.
Ex6	Exercising unconsciously	Whether or not a client unconsciously exercises more than enough owing to companionship during exercise performance.
Ex7	Receiving assessment	Whether or not a client is assessed about his/her health condition during ADC service use.
Ex8	Receiving treatment	Whether or not a client receives medical/nursing care during ADC service use.
Ex9	Receiving explanations about one’s health conditions	Whether or not a client receives explanations about his/her health condition, needs, and self-care during medical/nursing treatment in ADC agencies.
Ex10	Using one’s five senses	Whether or not a client tries to use his/her five senses in a more sensitive way within the group setting of the ADC compared to how he/she utilizes them in his/her usual home setting.
Ex11	Speaking aggressively	Whether or not a client speaks or tries to speak aggressively.
Ex12	Classifying ideas	Whether or not a client classifies or tries to classify the ideas that he/she wants to communicate.
Ex13	Recognizing value in helping others	Whether or not a client recognizes his/her value when he/she helps other people during ADC service use.
Ex14	Feeling like revisiting here	Whether or not a client feels like visiting the ADC agency again.
Ex15	Recognizing one’s own place in society	Whether or not a client feels comfortable when recognizing his/her place in society.
Ex16	Family member being away from the care recipient	Whether or not the client’s family caregiver spends his/her time free from the usual care they need to provide to the care recipient while the latter uses the ADC services.
Ex17	Family members being supported	Whether or not the client’s family caregiver is supported by ADC staff informationally and/or emotionally.

“Ex” means “Experience”.

**Table 2:** Frequency of clients’/family members’ experiences in adult day care service use.

	Mean (SD)	1 = Not at All		2 = Rarely		3 = Once every Two Visits		4 = Every Visit	
Ex1	3.57 (0.80)	15	(4.2)	24	(6.7)	62	(17.2)	258	(71.7)
Ex2	3.49 (0.86)	18	(5.0)	33	(9.2)	64	(17.8)	243	(67.5)
Ex3	2.46 (1.36)	146	(40.6)	38	(10.6)	36	(10.0)	136	(37.8)
Ex4	2.65 (1.32)	109	(30.3)	41	(11.4)	39	(10.8)	144	(40.0)
Ex5	3.53 (0.86)	19	(5.3)	30	(8.3)	52	(14.4)	259	(71.9)
Ex6	3.39 (0.96)	26	(7.2)	43	(11.9)	55	(15.3)	236	(65.6)
Ex7	3.14 (1.16)	47	(13.1)	79	(21.9)	9	(2.5)	224	(62.2)
Ex8	2.32 (1.30)	143	(39.7)	78	(21.7)	18	(5.0)	119	(33.1)
Ex9	2.28 (1.28)	142	(39.4)	89	(24.7)	15	(4.2)	114	(31.7)
Ex10	3.26 (0.98)	19	(5.3)	82	(22.8)	46	(12.8)	213	(59.2)
Ex11	3.33 (0.89)	11	(3.1)	70	(19.4)	70	(19.4)	209	(58.1)
Ex12	3.21 (1.04)	30	(8.3)	72	(20.0)	50	(13.9)	207	(57.5)
Ex13	2.96 (1.08)	40	(11.1)	95	(26.4)	60	(16.7)	162	(45.0)
Ex14	3.58 (0.79)	11	(3.1)	34	(9.4)	51	(14.2)	262	(72.8)
Ex15	3.58 (0.74)	2	(0.6)	48	(13.3)	48	(13.3)	261	(72.5)
Ex16	2.92 (1.33)	101	(28.1)	28	(7.8)	28	(7.8)	200	(55.6)
Ex17	2.08 (1.20)	160	(44.4)	91	(25.3)	26	(7.2)	82	(22.8)

Numbers are mean (standard deviation) or n (%).

**Table 3:** Results of principal component analysis.

		Factor 1	Factor 2	Factor 3	Factor 4
		Social Participation	Hygiene and Health	Exercise and Eating Habits	Family Support
Ex11		0.826			
Ex13		0.798			
Ex12		0.797			
Ex15		0.727			
Ex14		0.698			
Ex10		0.640			
Ex2		0.593			
Ex1		0.543		0.464	
Ex8			0.826		
Ex9			0.795		
Ex7			0.755		
Ex4			0.522		
Ex3			0.468		
Ex5				0.847	
Ex6				0.821	
Ex16					0.866
Ex17					0.815
**Sums of loading squares (%)**		25.4	15.9	14.1	10.4
**Cronbach’s alpha**		0.890	0.765	0.744	0.757

We used principal component analysis with varimax rotation to summarize the empirical dimensions of the items. Items were rated on a 4-item Liker-type scale: 1 = Not at all, 2 = Rarely, 3 = Once every two visits, 4 = Every visit. Loadings below 0.45 are omitted from the table.
